# Using digital notifications to improve attendance in clinic: systematic review and meta-analysis

**DOI:** 10.1136/bmjopen-2016-012116

**Published:** 2016-10-24

**Authors:** Dan Robotham, Safarina Satkunanathan, John Reynolds, Daniel Stahl, Til Wykes

**Affiliations:** 1 Institute of Psychiatry, Psychology & Neuroscience, King's College London (KCL), London, UK; 2 South London and Maudsley NHS Foundation Trust, London, UK

**Keywords:** Reminders, clinic attendance, no shows, healthcare efficiency

## Abstract

**Objectives:**

Assess the impact of text-based electronic notifications on improving clinic attendance, in relation to study quality (according to risk of bias), and to assess simple ways in which notifications can be optimised (ie, impact of multiple notifications).

**Design:**

Systematic review, study quality appraisal assessing risk of bias, data synthesised in meta-analyses.

**Data sources:**

MEDLINE, EMBASE, PsycINFO, Web of Science and Cochrane Database of Systematic Reviews (01.01.05 until 25.4.15). A systematic search to discover all studies containing quantitative data for synthesis into meta-analyses.

**Eligibility criteria:**

Studies examining the effect of text-based electronic notifications on prescheduled appointment attendance in healthcare settings. Primary analysis included experimental studies where randomisation was used to define allocation to intervention and where a control group consisting of ‘no reminders’ was used. Secondary meta-analysis included studies comparing text reminders with voice reminders. Studies lacking sufficient information for inclusion (after attempting to contact study authors) were excluded.

**Outcome measures:**

Primary outcomes were rate of attendance/non-attendance at healthcare appointments. Secondary outcome was rate of rescheduled and cancelled appointments.

**Results:**

26 articles were included. 21 included in the primary meta-analysis (8345 patients receiving electronic text notifications, 7731 patients receiving no notifications). Studies were included from Europe (9), Asia (7), Africa (2), Australia (2) and America (1). Patients who received notifications were 23% more likely to attend clinic than those who received no notification (risk ratio=1.23, 67% vs 54%). Those receiving notifications were 25% less likely to ‘no show’ for appointments (risk ratio=.75, 15% vs 21%). Results were similar when accounting for risk of bias, region and publication year. Multiple notifications were significantly more effective at improving attendance than single notifications. Voice notifications appeared more effective than text notifications at improving attendance.

**Conclusions:**

Electronic text notifications improve attendance and reduce no shows across healthcare settings. Sending multiple notifications could improve attendance further.

**Strengths and limitations of this study**

- Updates and appraises the evidence for how electronic text notifications impact on appointment attendance.
- Assesses study quality using a risk of bias framework.
- Large number of participants means that the impact of high quality studies can be considered.
- Tests the effect of multiple notifications, one way in which notifications can be optimised.

## Introduction

Reducing the number of missed healthcare appointments improves the efficiency of health services. Missing healthcare appointments without cancelling in advance results in a ‘no show’, a vacant appointment slot that cannot be offered to others. In 2015, the UK Secretary of State for Health estimated that missed general practitioner (GP) and hospital appointments cost the National Health Service (NHS) an estimated £912m per year^1^ and most appointments are missed due to simple reasons such as forgetfulness.^2^ ^3^ Missed appointments and no shows are more problematic in some areas of healthcare than others, for example, attendance is poor in community mental health settings which can have subsequent effects on care.^4^ ^5^

‘No shows’ can be reduced by reminding patients about their appointment in advance. The simplest way to do this is through electronic text notifications to patients' phones. Currently, there are as many mobile subscriptions as people in the world.^6^ In 2014, 93% of UK adults owned a mobile phone, with 61% having a smartphone (a 10% increase from 2013; Ofcom, 2014). In the last two decades, the service of sending text messages from mobile phones has dramatically changed the way in which people communicate. The number of messages sent has increased threefold to >150 billion between 2006 and 2011.^7^ This form of communication is acceptable to the public and has been harnessed by healthcare providers to remind patients about their appointments.

For the purposes of this review, the term ‘electronic text notifications’ refers to written messages sent from a service provider to a patient, in order to help patients remember, cancel or reschedule healthcare appointments. Notifications can be sent to patients' phones by text message, email or instant messaging applications. They cost little and can be delivered almost instantly.^8^ Unlike voice notifications, patients are able to reread and refer back to text notifications at their own convenience, and they may be perceived as less intrusive.^9^ They are used throughout the world across healthcare settings, with studies and reviews demonstrating increased appointment attendance.^10–13^ A recent meta-analysis and systematic review showed 50% improvements in attendance (relative to when no notification was provided);^14^ since this review was published, the use of technology is even more prevalent, with the use of smartphones almost doubling in the USA, (from 35% to 64% among adults).^15^

No-one has yet assessed the effectiveness of the intervention with regard to study quality/risk of bias. The large number of studies now available also allows an exploration of other potential predictive variables such as year of publication and geographical region. Similarly, there is little evidence on how to optimise electronic text notifications, specifically, whether the effect of multiple notifications is greater than the effect of a single notification and whether text notifications are as effective as voice notifications. This paper reviews and critically appraises the updated evidence for electronic text notifications and begins to answer such questions.

### Aim

This review explores how much electronic text notifications improve attendance at healthcare appointments.

## Methods

### Types of studies

We included all experimental studies containing quantitative data to be synthesised into a meta-analysis, in which randomisation was used to define allocation to the intervention. We included all studies published in the last 10 years (January 2005 until April 2015), including data from conference presentations where full published studies were unavailable. Where the same data had been published in two publications, the article with complete data was favoured (usually the later publication). Studies published prior to 2005 were excluded. The rationale is that mobile phone ownership was limited (and thus unrepresentative) prior to this date. No published protocol exists for this review.

### Types of participants

We included all participants in studies which contained data measuring the effect of electronic text notifications on scheduled appointment attendance in any healthcare setting.

### Types of interventions

We included studies examining the effect of electronic text notifications on the attendance of prescheduled healthcare appointments. Studies were only included in the primary analysis if they included a control group which received ‘no notifications’. In cases where studies had multiple comparison groups (eg, electronic text notifications vs voice notifications vs no notifications), the data from alternative intervention groups were included in a secondary analysis.

We excluded:

- Data relating to patients attending non-scheduled drop-in clinics or where patients were reminded to book future appointments, or health outcomes other than clinic attendance (eg, adherence to medication).
- Studies not published in the peer-reviewed literature or presented at academic conferences or which lacked sufficient information to be included in the meta-analysis after contacting study authors (ie, studies failing to report the number of patients allocated to receive an electronic text notifications intervention).

### Outcomes

Primary outcomes were the rate of attendance/non-attendance at healthcare appointments. The secondary outcome was the rate of rescheduling/cancellation of appointments (as opposed to ‘no show’ appointments, where the patient does not attend or cancel).

In addition to the effects on the primary and secondary outcomes, we investigated (i) whether potential predictive variables such as study quality, year of publication or geographical region affected the results, (ii) whether the number and timing of notifications affected the outcome, (iii) whether notifications had any effect in mental health settings which generally have the lowest attendance rates and (iv) how effective text reminders were in comparison to voice reminders (in studies which compared the two).

### Information sources

The following bibliographic databases were searched (25.4.15): MEDLINE, EMBASE, PsycINFO, Web of Science and The Cochrane Database of Systematic Reviews (from January 2005 until the search date). A hand search was also conducted of the reference lists of included studies, which identified two additional studies. The key terms used in the electronic searches for each of the databases are shown in online supplementary appendices 1–5. Authors of studies were contacted for further information when it was not present in the published data, for example, to clarify the patient groups they had included in their study.

10.1136/bmjopen-2016-012116.supp1 supplementary appendices

### Data collection process

Two reviewers (SS and JR) independently screened all the papers against the inclusion criteria. For the papers that met the inclusion criteria, the reviewers independently extracted information on the geographical location, clinic type, sample size, interventions and controls, study design, and comparison outcomes of attendance and non-attendance rates. Disagreements throughout this process were resolved by arbitration with a third reviewer (DR).

### Classification of data

Articles were included and interpreted based on the outcome measures used. This fell into three categories; attendance in clinic, ‘no show’ rates in clinic and cancellations/rescheduled appointments in clinic. Attendance rate and ‘no show’ rate were examined separately. Although there are many similarities between these outcomes, they cannot be assumed as equivalent as some unattended appointments may be cancelled or rescheduled in advance, in which case they are not classifiable as ‘no shows’. For those studies that measured attendance as a primary outcome measure, it was not possible to separate the proportion of ‘no shows’ and the proportion of cancellations.

### Assessment of risk of bias

Studies were appraised using Cochrane Handbook for Systematic Reviews of Interventions.^16^ For each study which was to be included in the primary analysis, two reviewers (SS and JR) independently assessed the risk of bias. Each domain was judged as ‘low’, ‘high’ or ‘unclear’ risk (when insufficient information was provided to permit judgement). The agreement rate between the raters was 79% (κ=0.58), a moderate level of agreement. Discrepancies between ratings were resolved by discussion with the third reviewer (DR).

All authors were contacted and asked for comments or clarifications on the risk of bias rating. The authors of 14 studies responded; changes were discussed with the reviewers. One or more changes were made to the ratings of seven studies. The most common reason for changing the rating was gaining access to study protocols. Studies were classified as either ‘at risk’ of bias or ‘at low risk’ of bias.

### Summary measures

The principle summary measure was risk difference between those who attended appointments compared with those who missed their appointment (expressed as a percentage); risk ratios were also calculated for the primary outcome. We compared those groups in which patients had received an electronic text notification with groups who received no notification.

We combined the results of participants receiving electronic text notification ‘intervention’ from all included studies. These were compared against ‘control’ participants who received no notification. The percentages of the primary outcome measure (attendance, ‘no shows’ and appointment cancellation) for all known intervention groups and control groups were extracted. Some studies presented data from multiple intervention groups (eg, from different clinics). In these cases, the intervention groups were the unit of analysis rather than the study itself, for example.^17^ A secondary meta-analysis pooled the data from studies comparing electronic text notifications against voice reminder notifications.

### Synthesis of results

Meta-analyses were conducted to determine the pooled effect size relating to intervention versus control groups using a random effects model. This is more realistic than fixed-effect meta-analyses in this situation due to the variety of populations and settings between studies.^18^ The primary meta-analysis was split by the three possible outcomes (i) attendance, (ii) ‘no shows’ and (iii) appointment cancellation. Pooled relative rates with exact Clopper-Pearson 95% confidence limits are presented. The risk ratio and the risk difference were calculated for included studies along with their CIs (at 95%), in order to calculate overall effect sizes for the intervention group and control group. Here, the risk ratio is the ratio of the probability of a positive event occurring in the intervention group to the probability of the event occurring in the control group. Interstudy heterogeneity was calculated using the I^2^ statistic (≥50% indicated heterogeneity). Heterogeneity was investigated by conducting meta-regressions to examine the influence of risk of bias, year of publication and geographical region. We also investigated the impact of multiple notifications in comparison to single notifications.

We investigated whether any study had a large influence on the pooled estimate in sensitivity analyses by re-estimating meta-analysis omitting each study in turn using Stata's (V.11.2, StataCorp, College Station, Texas, the USA) ‘metainf’ command.^19^

Publication bias was assessed by visual inspections of funnel plots, Egger's test and using a non-parametric ‘trim and fill’ method.^20^ If the conclusion of the meta-analysis remains unchanged following adjustment for the publication bias using the trim and fill method, the results can be considered as robust, excluding publication bias. All analyses were carried out in Stata (V.11.2, StataCorp).

## Results

### Study selection

**Figure 1:**
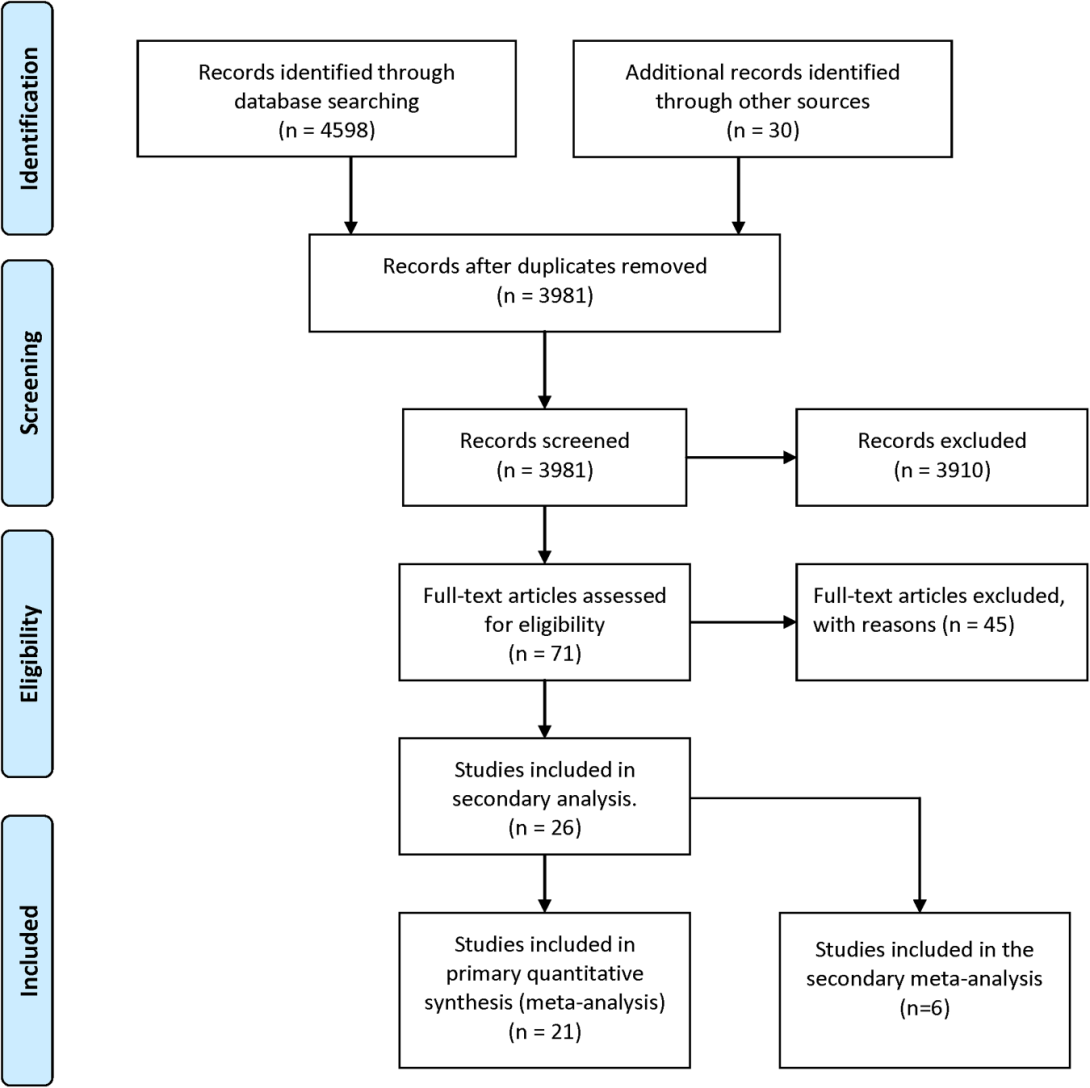
PRISMA flow chart. PRISMA, Preferred Reporting Items for Systematic review and Meta-Analysis.

After duplicates had been removed, 3981 articles were screened. Of these, 3910 articles were excluded based on the abstract alone; these studies clearly did not meet the inclusion criteria. A further 45 articles were excluded after reading full-text articles. The primary reason for exclusion in each case were as follows: non-randomised study (n=23), examined the effects of electronic text notifications on reminding patients to take medication or to make appointments (rather than to attend) (n=3), only collecting data on preference for notifications rather than information about their effect on attendance (n=9), only providing secondary data or where the same data had been assimilated in another included paper (n=6), exclusively using other notification systems such automated call backs (n=2) or lacking sufficient information to be included in the meta-analysis after we attempted to contact authors (n=2). In total, 26 articles met the study inclusion criteria and were included in the systematic review. Of these, five studies were excluded from the primary meta-analysis because they lacked a ‘no intervention’ control group. Instead, they were included in the secondary meta-analysis comparing electronic notifications against voice notifications. The Preferred Reporting Items for Systematic review and Meta-Analysis (PRISMA) flow chart (figure 1)^21^ describes the process in which studies were included and excluded:

### Study characteristics for primary meta-analysis

Of all data included in the primary meta-analysis, 12 studies used attendances as an outcome measure, 16 measured ‘no show’ rates and 3 measured cancellation. A total of 8345 patients received electronic text notifications, and 7731 patients received no notification. Randomised studies typically compared attendance or ‘no show’ rates of those who received electronic text notifications with those who did not receive notifications.

Studies spanned Europe (nine), Asia (seven), America (one), Africa (two) and Australia (two). The most common study context was primary care/general physical healthcare (11); followed by sexual health (3), dental care (2), mental health (2), and paediatrics (2). Other types of health intervention included paediatrics, postnatal care, blood donation, optometry, chronic illness, allergies and mixed contexts.

All included studies used short message service (SMS) notifications; one study used this in conjunction with follow-up phone calls and postal notifications. A typical example of an electronic text notification informed the patient of the time and date of the appointment and asked them to respond if they could not come to the appointment, for example: ‘You have an appointment on… (date) at … (time) with Dr … (name) Please answer NO if you do not intend to come’.

Studies differed in the number and in the timing of notifications sent to patients. The majority of studies (n=13) sent only one notification. Two notifications were sent in two studies; more than two notifications were sent in three studies, one study used voice notifications followed up with electronic text notifications (this was classified as a study in which ‘more than one notification’ was sent). One study did not provide this information. In one anomalous study, notifications were sent on every day for 30 days prior to an appointment. Regarding timing of messages, in nine studies notifications were sent 48 hours (or less) before the appointment. In three studies, notifications were sent over 48 hours before the appointment. In one study, participants were sent a notification 8 weeks prior to their appointment. Of the eight studies where two or more notifications were sent, the majority (n=5) reminded patients before and after the 48 hour mark. Full details of individual studies are presented in table 1.

**Table 1:** Studies included in meta-analyses

Study	Subject area; study design; country	Participants	Intervention and comparator	Notification characteristics	Outcome measures	Prevalence rates
Arora *et al*^22^ (2015)	Primary care; randomised; the USA	In total, 328 patients from the Emergency Department at Los Angeles County USC Medical Center.	*Interventions*: SMS reminders *Control*: no reminders	Three reminders: 7, 3 and 1 days before appointment.	Attendance rate	Int=73% Control=62%
Bigna *et al*^23^ (2014)	Preventative medicine; randomised; Cameroon	In total, 242 adult–child (carer patient) pairs who were infected with or had been exposed to HIV attending clinics in urban, semiurban and rural settings.	*Intervention*: SMS reminders *Control***:** no reminders	One reminder: 2 or 3 days before appointment	Attendance rate	*Attendance* Int=75% Control=51%
Bos *et al*^24^ (2005)	Dental care; randomised; the Netherlands	In total, 143 patients attending the orthodontic department of the Academic Centre of Dentistry in Amsterdam.	*Intervention*: SMS reminders *Control***:** no reminders	One reminder: 1 day before appointment	Attendance rate, non-attendance rate, cancellation/reschedule rate	*Attendance* Int=3% Control=7% ‘*No show’* Int=82% Control=84% *Cancellations* Int=16% Control=10%
Chen *et al*^25^ (2008)	Health promotion; randomised; China	In total, 1891 adults who had scheduled appointments within 72 hours to 2 months from recruitment.	*Intervention***:** SMS reminders *Control***:** no reminders	One reminder: 3 days before appointment	Attendance rate	Int=88% Control=81%
Cho *et al*^26^ (2010)	Health promotion; randomised; South Korea	In total, 918 adults attending family practices for lipid lowering.	*Intervention***:** SMS reminders *Control*: no reminders *Note*: a third group of participants received postal reminder (not included)	One reminder: 8 weeks before appointment (week 16 of 24).	Attendance rate	Int=76% Control=72%
Clough and Casey^27^ (2014)	Mental health; Randomised; Australia	140 consecutive adults seeking psychotherapeutic treatment at an outpatient psychology clinic at Brisbane University.	*Intervention*: SMS reminders *Control*: No reminders	One reminder: 1 day before appointment (between 8:30 and 9:00)	Attendance rate, Non-attendance rate, Cancellation/reschedule rate	*Attendance* Int=89% Control=91% *‘No show’* Int=7% Control=6% *Cancellations* Int=4% Control=3%
Costa *et al*^28^ (2008)	General health; randomised; Portugal	In total, 3362 patients of San Sebastião Hospital who had mobile phone number registered to the system.	*Intervention*: SMS reminders *Control*: no reminders	One reminder: 2 working days before appointment	Non-attendance rate	Int=10% Control=13%
Fairhust and Sheikh^29^ (2008)	General health; randomised; UK (Scotland)	In total, 415 appointments made by 173 patients who had failed to attend two or more appointments the year before.	*Intervention*: SMS reminders *Control*: no reminders	One reminder: half a day before appointment (between 8:00 and 9:00, before afternoon appointments, or 14:00 to 15:00 the day before morning appointments).	Non-attendance rate	Int=12% Control=17%
Koury and Faris^30^ (2005)	ENT; Randomised; UK	In total, 291 patients who were scheduled for an otolaryngology outpatient clinic at a UK district general hospital.	*Intervention*: SMS reminders *Control*: no reminders	No further details	Non-attendance rate	Int=6% Control=14%
Leong *et al*^31^ (2006)	Primary care; randomised; Malaysia	In total, 664 patients from seven primary care clinics whose follow-up appointments fell between 48 hours and 3 months from recruitment date.	*Intervention*: SMS reminders *Control*: no reminders	One reminder: 24–48 hours before appointment	Attendance rate	Int=60% Control=48%
Liew *et al*^32^ (2009)	Chronic illnesses; randomised; Malaysia	In total, 617 patients requiring chronic disease care from two primary care clinics in Kuala Lumpur.	*Intervention*: SMS reminders *Control***:** No reminders *Note*: a third group of participants received telephone reminder (included in secondary analysis)	One reminder: 24–48 hours before appointment	Non-attendance rate	Int=16% Control=23%
Lin *et al*^33^ (2012)	Paediatric clinic/ophthalmology; randomised; China	In total, 258 parent/child pairs were randomised. Children required treatment for cataracts.	*Intervention*: SMS reminders to parents *Control*: no reminders	Four reminders: Two each at 4 days and 1 day before appointment (at 10:00 and 4:00) Each patient was sent reminders in advance of four separate appointments.	Attendance rate	Int=91% Control=62%
Narring *et al*^34^ (2013)	Youth clinic; randomised; Switzerland	In total, 616 patients aged 12–24 years with primary care appointments at a multidisciplinary clinic. Plus 203 patients with gynaecological appointments and 165 patients with mental healthcare appointments at a multidisciplinary clinic.	*Intervention*: SMS reminders *Control***:** no reminders	One reminder: 8:00–11:00 the day before appointment	Non-attendance rate	Int=20% Control=20%
Odeny *et al*^35^ (2012)	Sexual health; randomised; Kenya	In total, 1188 men undergoing circumcision at any of 12 sites in Nyanza province.	*Intervention*: SMS reminders *Control***:** no reminders	Seven reminders: daily for 7 days before appointment	Non-attendance rate	Int=35% Control=40%
Perron *et al*^36^ (2010)	Sexual health; randomised; Switzerland	In total, 2123 patients scheduled to attend primary care clinic and ambulatory HIV clinic of the Geneva University Hospitals, between April and June 2008	*Intervention*: a combination of phone, SMS and postal reminders *Control*: no reminders	Sequential intervention, one phone call 48 hours before appointment. If phone was not answered after three attempts, either text message sent or postal message if they did not have a phone.	Non-attendance rate	Int=8% Control=11%
Prasad and Anand^37^ (2012)	Dental care; randomised; the Netherlands	In total, 206 patients who were scheduled to attend four selected departments from September 2010 to December 2010	*Intervention*: SMS reminders *Control*: no reminders	Two reminders: 24 hours before, and on day of appointment	Attendance rate	Int=79% Control=36%
Reeve-Mates *et al* (under review)	Mental health, randomised, UK	In total, 75 patients attending mental health services from January to July 2014.	*Intervention*: SMS reminders *Control***:** no reminders	Two reminders: 7 days and 1 day before appointment	Attendance rate, non-attendance rate, cancellation/reschedule rate	*Attendance* Int=70% Control=59% ‘*No show’* Int=6% Control=20% *Cancellations* Int=15% Control=13%
Rutland *et al*^38^ (2012)	Sexual health; randomised; UK	In total, 252 patients aged 16–30 years who booked an appointment during the 6 month study period. Only gave intervention to people who had missed appointments in the past.	*Intervention 1*: SMS reminders *Intervention 2*: SMS reminders plus health promotion *Control*: no reminders	One reminder: 1 week after they had missed their initial appointment (for attendance within 4 weeks).	Non-attendance rate	(Pooled) Int=12% Control=5%
Taylor *et al*^39^ (2012)	Physical therapy; randomised; Australia	In total, 696 participants who had an appointment in a physical therapy outpatient clinic at one of the two participating clinics.	*Intervention*: SMS reminders *Control*: no reminders	One reminder: 2 days before appointment (if made 3+ days prior), 1 day before appointment (if made 2 days prior).	Non-attendance rate	Int=11% Control=16%
Wang *et al*^40^ (2014)	Allergic rhinitis; randomised; China	In total, 50 patients with a history of physician-diagnosed allergic rhinitis who had an appointment scheduled from December 2011 to March 2012.	*Intervention*: SMS reminders *Control***:** no reminders	A total of 30 reminders for medication: daily reminder for 30 days, at 7:00 on Monday to Friday and at 9:00. on Saturday and Sunday.	Attendance rate	Int=72% Control=40%
Youssef, *et al*^17^ (2014)	General health; randomised; Saudi Arabia	In total, 2297 outpatients attending one of four clinics at the King Fahad teaching hospital from April to June 2011.	*Intervention*: SMS reminders *Control*: no reminders	One reminder: 48 hours before appointment.	Non-attendance rate	(Pooled) Int=27% Control=37%
Studies included in secondary meta-analysis						
Fung *et al*^41^ (2009)	Blood donor; randomised; the USA	In total, 31 repeat blood donors who made donation appointments in October 2008.	*Intervention 1***:** SMS reminders *Intervention 2***:** telephone reminders	No information available	Attendance rate	Int=56%
Liew *et al* (2009) (also included in primary anlaysis)	Chronic illnesses; randomised; Malaysia	In total, 617 patients requiring chronic disease care from two primary care clinics in Kuala Lumpur.	*Intervention 1*: SMS reminders *Intervention 2***:** telephone reminders *Control***:** no reminders (included in primary meta-analysis)	One reminder: 24–48 hours before appointment	Non-attendance rate	Int=16% Control=23%
Nelson *et al*^42^ (2011)	Paediatric dentistry; randomised; the USA	In total, 318 caregiver/child dyads attending a paediatric dentistry clinic at the University of Washington, Seattle.	*Intervention 1***:** SMS reminders *Intervention 2***:** telephone reminders	One reminder: 48 hours before appointment	Attendance rate Non-attendance rate	*Attendance* Int=82.3% ‘*No show’* Int=17.7%
Norton *et al*^43^ (2014)	Sexual health; randomised; the USA	In total, 52 adults from the Duke University Medical Center (Durham, NC) Adult Infectious Diseases Clinic from June to August 2010.	*Intervention 1***:** SMS reminders *Intervention 2***:** telephone reminders	One reminder: 1 day before appointment	Attendance rate	Int=72%
Percac-Lima *et al*^44^ (2014)	Primary care; randomised; the USA	In total, 7488 outpatients from Massachusetts General Hospital Chelsea Healthcare Centre.	*Intervention 1***:** SMS reminder *Intervention 2*: telephone reminders	Two reminders: 7 days and 1 day before appointment	Non-attendance rate	Int=18%
Perron *et al*^45^ (2013)	General health; randomised; Switzerland	In total, 3585 patients attending Geneva University Hospitals from November 2010 to April 2011.	*Intervention***:** SMS reminders *Intervention 2*: telephone reminders	One reminder: 24 hours before appointment	Non-attendance rate	Int=12%

ENT, ear nose throat; Int, intervention; SMS, short message service.

### Study characteristics for secondary meta-analysis

The secondary meta-analysis included six studies (only one of which had been included in the primary meta-analysis). Attendance rates were measured in three studies, ‘no show’ rates were measured in four. A total of 9885 patients received electronic text notifications, and 5076 patients received voice notifications. The studies were conducted in America (four), Europe (one) and Asia (one). Context included were as follows: primary care/general healthcare (two), sexual health (one), dental paediatrics (one), blood donation (one) and chronic illness (one).

### Risk of bias within and across studies

The risk of bias within individual studies is presented in online supplementary appendix 6. We used items of Cochrane's framework in judging the quality of the studies. The corresponding author of each article was sent their assessment to check and suggest revisions if necessary. Biases relating to blinding were considered of lesser importance in context of the intervention; participants cannot be blinded to a notification intervention, and outcome assessment is objective (ie, the participant either attended appointment or not). Random sequence generation and incomplete outcome data were considered important potential biases. The most common reason for ‘unclear’ bias was the unavailability of protocols.

### Primary meta-analysis results: main outcomes

**Figure 2:**
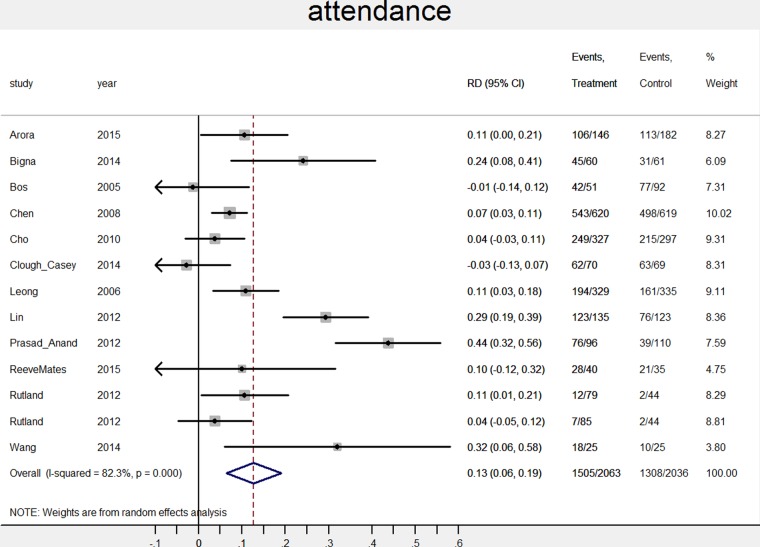
Effect of notifications on attendance rates. RD, risk difference.

**Figure 3:**
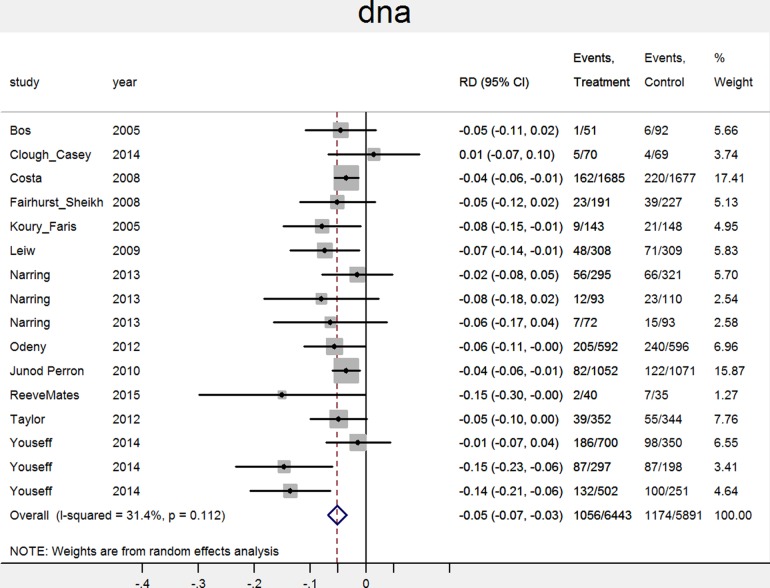
Effect of notifications on ‘no show’ rates. RD, risk difference.

The pooled attendance rate was 67% (N=13, CI 53% to 82%) for intervention groups and 54% (N=13, CI 37% to 70%) for control groups. The risk ratio was 1.23 (CI 1.10 to 1.38; N=13, p<0.01, I^2^=83%), the risk difference was 13% in favour of the intervention group (95% CI 6% to 19%; N=13, p<0.01, I^2^=82%). The pooled ‘no show’ rate was 15% (N=16, CI 10% to 19%) for intervention groups and 21% (N=16, CI 16% to 26%) for control groups. The risk ratio was 0.75 (CI 0.68 to 0.82; N=16, p<0.01, I^2^=21%), the risk difference was 5% in favour of the intervention group (95% CI −7% to −3%; N=16, p<0.01, I^2^=31%). The percentage difference between intervention and control groups for each study is shown in figures 2 and 3.

The pooled cancellation rates were 11% for intervention (N=3, CI −2% to 19%) and 8% control (N=3, CI −1% to 14%) groups. The risk ratio was non-significant at 1.37 (N=3, p=0.34, I^2^<1) as was the 2% risk difference at (N=3, p=0.4, I^2^<1).

**Figure 4:**
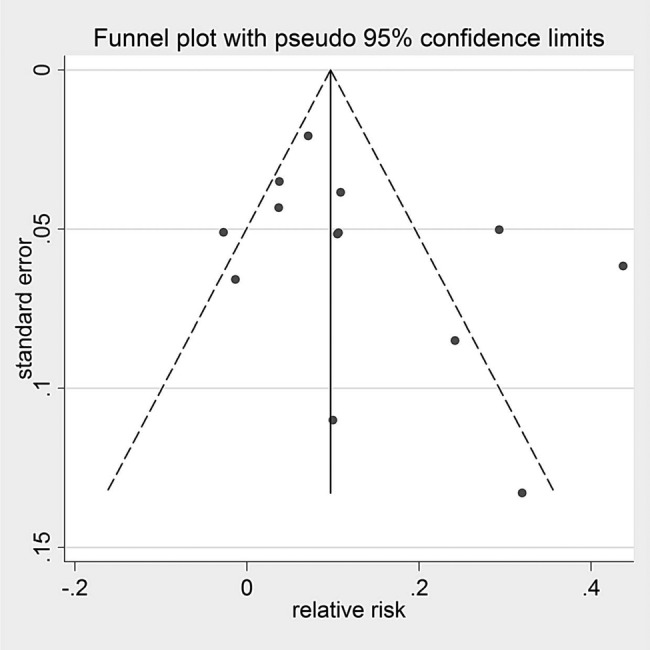
Funnel plot for attendance rates.

**Figure 5:**
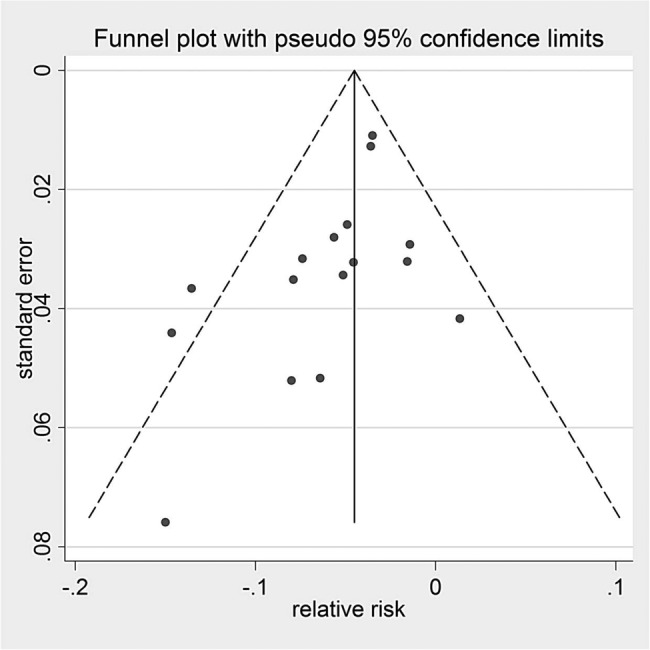
Funnel plot for ‘no show’ rates.

Visual inspection of funnel plots (see figures 4 and 5, see online supplementary appendix 7) revealed evidence of potential publication bias in attendance but little evidence of publication bias in ‘no shows’. The trim and fill method revealed no missing studies. Egger's test was not significant in the meta-analyses.

### Assessing and identifying study heterogeneity

Meta-regression compared the impact of the following potential predictive variables: risk of bias (high bias, low bias), number of notifications (one, multiple), year of publication (2005–2010, 2011–2015) and geographic region (Europe, Asia, other), where the ‘other’ category was created because there were few studies in the other continents.

Of these variables, the only significant finding was the effect of multiple reminders on appointment attendance (shown in table 2). Multiple notifications increased the risk of patients attending appointments by 25% (compared with 6% for patients receiving one notification), but multiple reminders did not make a significant difference in reducing ‘no shows’. No significant effects were found for risk of bias (p=0.88 for attendance, p=0.68 for ‘no shows’), age of study (p=0.16 for attendance, p=0.38 for ‘no shows’) or geographic region (F=0.11, p=0.9 for attendance; F=1.6, p=0.23 for ‘no shows’). No significant associations were found when all variables were pooled (F=2.35 p=0.15 for attendance; F=0.66, p=0.66 for ‘no shows’).

**Table 2:** Effect of notification frequency

	Intervention (%)	Control (%)	Risk ratio	Risk difference (%)	Coeff* (%)	p value (risk difference)	I^2^ (Risk difference) (%)	Obs
*Attendance*								
More than one notification	78 (N=5, CI 68 to 88)	52 (N=5, CI 31 to 78)	1.49 (CI 1.17 to 1.88)	25 (CI 11 to 39)	19	0.01	66	13
One notification	62 (N=8, CI=40 to 83)	55 (N=8, CI=31 to 78)	1.09 (CI=1.00 to 1.18)	6 (CI=2 to 10)				
*‘No show’ rate*								
More than one notification	16 (N=3, CI −3 to 3)	24 (N=3, CI 2 to 46)	0.75 (CI 0.57 to 0.99)	−5 (CI=−8 to −1)	0.3	0.91	35	15
One notification	15 (N=12, CI 10 to 20)	21 (N=12, CI 15 to 26)	0.75 (CI 0.68 to 0.82)	−5 (CI −8 to −3)				

Obs, number of observations.

*Coeff. presents difference in % risk difference between more than one notification and one notification.

### Sensitivity analyses

In a sensitivity analysis in which the meta-analyses were repeated, excluding one study at a time to investigate the influence of each individual study on the overall meta-analysis summary did not reveal that any single study significantly affected the results.

### What happens in mental health?

We pooled the results of three studies (182 participants received notifications, 197 did not). Two studies measured attendance and three measured ‘no shows’. One took place in psychosis services, one in a university therapeutic context and one at a youth clinic. The pooled attendance rates were 85% for intervention (N=2, CI 78% to 91%) and 87% for control (N=2, CI 81% to 93%). The risk ratio was 1.01 (CI 0.85 to 1.2; N=2, p=.92, I^2^=30%), the risk difference was <1% (95% CI −11% to 12%; N=2, p=0.93, I^2^=25%). The pooled rate of ‘no shows’ was 7% for intervention (N=3, CI 3% to 11%) and 13% for control (N=3, CI 4% to 22%). The risk ratio was 0.61 (CI 0.29 to 1.29; N=3, p=0.2, I^2^=21%), the risk difference was 5% (95% CI −14% to 4%; N=3, p=0.26, I^2^=52%).

### How do electronic text notifications compare to voice notifications? (secondary meta-analysis)

The pooled attendance rate for the electronic text notifications was 74% (N=3, CI 60% to 88%) and 74% for voice notifications (N=3, CI 51% to 97%). This difference was significant; however, the risk ratio was 0.90 (CI 0.82 to 0.98; N=3, p=0.01, I^2^<1%), the risk difference was 8% in favour of voice notifications (95% CI −16% to 0.1%; N=3, p=0.05, I^2^=6%). Pooled ‘no show’ rates were 15% for electronic text notifications (N=4, CI 11% to 20%) and 13% for voice notifications (N=4, CI 7% to 18%). The risk ratio was 1.12 (CI 0.90 to 1.38; N=4, p=0.32, I^2^<73%), the risk difference was 1% (95% CI −2% to 4%; N=4, p=0.35, I^2^=70%).

## Discussion

This review and meta-analysis demonstrates that electronic text notifications improve appointment attendance and reduce ‘no shows’. Notifications improve attendance and reduce ‘no shows’. These findings replicate earlier ones,^14^ but we can have more confidence in the results because they were stable even after removing the influence of studies which were at risk of bias. A novel finding is that two or more notifications increased attendance by as much as 19% over and above sending one notification, and voice notifications may offer slight improvements over text notifications for increasing attendance.

Taking the UK Secretary of State's estimates literally, a 5% reduction in ‘no shows’ across the National Health Service (NHS) GPs and hospitals would save the NHS >£45 million. There may be additional savings gained by sending multiple (as opposed to single) notifications. Almost all NHS services have an electronic text notification system already; these could be adapted to provide an extra notification at little extra cost to accommodate this change.

Some areas, such as mental health, have historically reported high rates of missed appointments,^5^ where people with severe mental illness may miss up to 45% of scheduled appointments in primary care.^46^ The studies reviewed here suggested that attendance rates for mental health settings were not dissimilar to those in other settings. These studies do not, therefore, reflect the ‘normal’ clinic attendance known to be lower and therefore suggests that more studies reflecting usual practice are needed. For those clinical areas with poor attendance, text messages may not act in the same way and may need to be adapted. But currently, we do not have any evidence to draw any conclusion.

### Strengths and limitations

The main strength of this review is the synthesis of large data sets. We were able to distinguish between different types of outcomes (attendance and ‘no shows’). The study provides basic information about how to optimise notifications, but more is needed in this area.

### Future research directions

This review confirms the importance of electronic text notifications in healthcare. To make the notifications even more effective we need to examine the tone and content of notifications, and whether the effects differ between client groups, and what preferences patients have for receiving notifications.

## Conclusions

Electronic text notifications increase attendance and reduce ‘no shows’. Multiple notifications add significantly to the effectiveness. The large number of ‘no shows’ in health services means any successful intervention to reduce them will have cost implications.
