# Supplementary material for: Using digital notifications to improve attendance in clinic: systematic review and meta-analysis
Source: BMJ Open. 2016 Oct 24;6(10):e012116. doi: 10.1136/bmjopen-2016-012116 (PMC5093388; doi:10.1136/bmjopen-2016-012116)
Supplement: supplementary appendices [file bmjopen-2016-012116supp_appendices.pdf]

Appendices

Searched Databases:
- Embase: embase 1980 to april 2015 week 15.
- Medline: 1946 to april 2015 week 2.
- Psychinfo: 1806 to April Week 2 2015.
- Web of science searched 22nd April 2015.
- Cochrane Database of Systematic Reviews : Issue 4 of 12, April 2015
- Cochrane Central Register of Controlled Trials : Issue 3 of 12, March 2015

Appendix 1. MEDLINE (Ovid) search strategy:

1. cellular phone/
2. ((cell* or mobile or wireless) adj (phone* or telephon*)).tw.
3. (cellphone* or mobiles or mhealth or m-health).tw.
4. ((mobile or handheld or hand-held) adj2 (device* or technolog* or app* or health*)).tw.
5. (smart phone* or smartphone* or blackberry or iphone* or android phone* or google android or ipod touch or personal digital assistant* or pda or pdas).tw.
6. 1 or 2 or 3 or 4 or 5
7. (text* or messag* or multimedia or multi-media or imag* or mms or data or input* or application* or app?).tw.
8. 6 and 7
9. text messaging/
10. ((text or short or multimedia or multi-media) adj1 messag*).tw.
11. sms.tw.
12. (texting* or texted or texter*).tw.
13. (mms and (multimedia or multi-media or messag*)).mp.
14. Electronic mail/
15. (electronic adj3 mail$).ab,ti.
16. (electronic adj3 messag$).ab,ti.
17. (email$ or e-mail$).ab,ti.
18. (noti* adj6 (patient* or client* or service-user* or people)).ti,ab,kw.
19. ((remind* or alert* or return* or fail*) adj2 (patient* or client* or service-user* or people)).tw.
20. ((appointment* or attend* or arriv* or consul*) adj2 (patient* or client* or service-user* or people)).tw.
21. (non?attend or non attend* or no show).ti,ab,kw.
22. ((appointment* or attend*) adj6 (complicance or fail* or keep* or miss* or prompt*)).ti,ab,kw.
23. exp Reminder Systems/
24. exp "Appointments and Schedules"/
25. exp Patient Compliance/
26. Outpatients/
27. Outpatient Clinics, Hospital/ut [Utilization]
28. Case Management/
29. Office Visits/
30. 8 or 9 or 10 or 11 or 12 or 13 or 14 or 15 or 16 or 17 or 18
31. 19 or 20 or 21 or 22 or 23 or 24 or 25 or 26 or 27 or 28 or 29
32. 30 and 31
33. exp animals/ not humans.sh.
34. 32 not 33
35. limit 34 to yr="1993 -Current"

Appendix 2. EMBASE (Ovid) search strategy:

1. mobile phone/
2. ((cell* or mobile or wireless) adj (phone* or telephon*)).ti,ab,kw.
3. (cellphone* or mobiles or mhealth or m-health).ti,ab,kw.
4. ((mobile or handheld or hand-held) adj2 (device* or technolog* or app* or health*)).ti,ab,kw.
5. personal digital assistant/
6. (smart phone* or smartphone* or blackberry or iphone* or android phone* or google android or ipod touch or personal digital assistant* or pda or pdas).ti,ab,kw.
7. 1 or 2 or 3 or 4 or 5 or 6
8. (text* or messag* or multimedia or multi-media or imag* or mms or data or input* or application* or app?).ti,ab,kw.
9. 7 and 8
10. text messaging/
11. ((text or short or multimedia or multi-media) adj1 messag*).ti,ab,kw.
12. sms.ti,ab,kw.
13. (texting* or texted or texter*).ti,ab,kw.
14. (mms and (multimedia or multi-media or messag*)).ti,ab,kw.
15. e-mail/
16. (electronic adj3 mail$).ti,ab,kw.
17. (electronic adj3 messag$).ti,ab,kw.
18. (email$ or e-mail$).ti,ab,kw.
19. (noti* adj6 (patient* or client* or service-user* or people)).ti,ab,kw.
20. 9 or 10 or 11 or 12 or 13 or 14 or 15 or 16 or 17 or 18 or 19
21. exp reminder system/
22. hospital management/
23. exp patient compliance/
24. exp outpatient care/
25. ((remind* or alert* or return* or fail*) adj2 (patient* or client* or service-user* or people)).ti,ab,kw.
26. ((appointment* or attend* or arriv* or consul*) adj2 (patient* or client* or service-user* or people)).ti,ab,kw.
27. (non?attend or non attend* or no show).ti,ab,kw.
28. ((appointment* or attend*) adj6 (complicance or fail* or keep* or miss* or prompt*)).ti,ab,kw.
29. 21 or 22 or 23 or 24 or 25 or 26 or 27 or 28
30. 20 and 29

Appendix 3. PsycINFO (Ovid) search strategy:

1. Cellular Phones/
2. ((cell* or mobile or wireless) adj (phone* or telephon*)).ti,ab,id.
3. (cellphone* or mobiles or mhealth or m-health).ti,ab,id.
4. ((mobile or handheld or hand-held) adj2 (device* or technolog* or app* or health*)).ti,ab,id.
5. Mobile Devices/
6. (smart phone* or smartphone* or blackberry or iphone* or android phone* or google android or ipod touch or personal digital assistant* or pda or pdas).ti,ab,id.
7. 1 or 2 or 3 or 4 or 5 or 6
8. (text* or messag* or multimedia or multi-media or imag* or mms or data or input* or application* or app?).ti,ab,id.
9. 7 and 8
10. electronic communication/
11. ((text or short or multimedia or multi-media) adj1 messag*).ti,ab,id.
12. sms.ti,ab,id.
13. (texting* or texted or texter*).ti,ab,id.

14. (mms and (multimedia or multi-media or messag*)).ti,ab,id.
15. exp computer mediated communication/
16. (electronic adj3 mail$).ti,ab,id.
17. (electronic adj3 messag$).ti,ab,id.
18. (email$ or e-mail$).ti,ab,id.
19. (noti* adj6 (patient* or client* or service-user* or people)).ti,ab,id.
20. 9 or 10 or 11 or 12 or 13 or 14 or 15 or 16 or 17 or 18 or 19
21. exp treatment compliance/
22. exp outpatient treatment/
23. treatment dropouts/
24. exp client attitudes/
25. ((remind* or alert* or return* or fail*) adj2 (patient* or client* or service-user* or people)).ti,ab,id.
26. ((appointment* or attend* or arriv* or consul*) adj2 (patient* or client* or service-user* or people)).ti,ab,id.
27. (non?attend or non attend*).ti,ab,id.
28. ((appointment* or attend*) adj6 (complicance or fail* or keep* or miss* or prompt*)).ti,ab,id.
29. 21 or 22 or 23 or 24 or 25 or 26 or 27 or 28
30. 20 and 29

Appendix 4. WEB of Science search strategy:

1. **TS=((cell* or mobile or wireless) NEAR/1 (phone* or telephon*))**
2. TS=(cellphone* or mobiles or mhealth or m-health)
3. TS=((mobile or handheld or hand-held) NEAR/2 (device* or technolog* or app* or health*))
4. TS=(smart phone* or smartphone* or blackberry or iphone* or android phone* or google android or ipod touch or personal digital assistant* or pda or pdas)
5. #4 OR #3 OR #2 OR #1
6. TS=(text* or messag* or multimedia or multi-media or imag* or mms or data or input* or application* or app?)
7. #6 AND #5
8. TS=((text or short or multimedia or multi-media) NEAR/1 messag*)
9. TS=(texting* or texted or texter* or sms)
10. TS=(mms and (multimedia or multi-media or messag*))
11. TS=(electronic NEAR/3 mail$)
12. TS=(electronic NEAR/3 messag$)
13. TS=(email$ or e-mail$)
14. TS=(noti* NEAR/6 (patient* or client* or service-user* or people))
15. #14 OR #13 OR #12 OR #11 OR #10 OR #9 OR #8 OR #7
16. TS=((remind* or alert* or return* or fail*) NEAR/2 (patient* or client* or service-user*))
17. TS=((appointment* or attend* or arriv* or consul*) NEAR/2 (patient* or client* or service-user*))
18. TS=(non?attend or “non attend*” or “no show”)
19. TS=((appointment* or attend*) NEAR/6 (complicance or fail* or keep* or miss* or prompt*))
20. #19 OR #18 OR #17 OR #16
21. #20 AND #15

Appendix 5. (Cochrane database of systematic reviews) & Cochrane Central Register of Controlled Trials search Strategy:

1. ((cell* or mobile or wireless) adj (phone* or telephon*))
2. (cellphone* or mobiles or mhealth or m-health)

3. ((mobile or handheld or hand-held) adj2 (device* or technolog* or app* or health*))
4. ("smart phone*" or smartphone* or blackberry or iphone* or "android phone*" or "google android" or ipod touch or "personal digital assistant*" or pda or pdas)
5. #1 or #2 or #3 or #4
6. (text* or messag* or multimedia or multi-media or imag* or mms or data or input* or application* or app?)
7. #5 and #6
8. ((text or short or multimedia or multi-media) adj messag*)
9. (texting* or texted or texter* or sms)
10. (mms and (multimedia or multi-media or messag*))
11. (electronic adj mail$)
12. (electronic adj3 messag$)
13. (email$ or e-mail$)
14. (noti* adj6 (patient* or client* or service-user* or people))
15. #7 or #8 or #9 or #10 or #11 or #12 or #13 or #14
16. ((remind* or alert* or return* or fail*) adj2 (patient* or client* or service-user* or people))
17. ((appointment* or attend* or arriv* or consul*) adj2 (patient* or client* or service-user* or people))
18. (non?attend or "non attend*" or “no show”)
19. ((appointment* or attend*) adj6 (complicance or fail* or keep* or miss* or prompt*))
20. #16 or #17 or #18 or #19
21. #15 and #20

Appendix 6: Cochrane Risk of Bias Summary for individual studies

	**Random Sequence Generation**	**Allocation Concealment**	**Blinding of Outcome Assessment**	**Incomplete Outcome Data**	**Selective Reporting**	**Other Bias**	**At high risk of bias?**
Arora et al. (2015) (22)	L	L	L	L	U	L	**N**
Bigna et al. (2014) (23)	L	L	L	L	L	U	**N**
Bos et al. (2005) (24)	H	U	L	U	U	H	**Y**
Chen et al. (2008) (25)	L	U	L	L	U	U	**N**
Cho et al (2010) (26)	L	L	L	L	U	L	**N**
Clough & Casey (2014) (27)	L	H	L	L	U	L	**N**
Costa et al (2008) (28)	U	U	L	U	U	U	**Y**
Fairhurst & Sheikh (2008) (29)	L	L	L	L	L	H	**N**
Koury & Faris (2005) (30)	U	U	U	U	U	U	**Y**
Leong et al. (2006) (31)	L	H	L	L	L	H	**N**
*Liew et al. (2009) (32)	L	L	L	L	U	H	**N**
Lin et al (2012) (33)	L	L	L	L	U	H	**N**
Narring et al. (2013) (34)	L	U	L	L	U	L	**N**
Odeny et al. (2012) (35)	L	L	L	L	U	L	**N**
Perron et al. (2010) (36)	L	U	L	L	U	L	**N**

Prasad & Anand (2012) (37)	U	U	L	L	U	H	**Y**
Reeve Mates et al (under review)	L	U	L	L	U	L	**N**
Rutland et al. (2012) (38)	U	U	U	U	U	U	**Y**
Taylor et al. (2012) (39)	L	L	L	L	L	L	**N**
Wang et al. (2014) (40)	L	L	L	L	U	L	**N**
Youssef, et al. (2014) (17)	L	L	L	L	L	L	**N**
Included in secondary meta-analysis only							
Fung et al.(2009) (41)	U	U	U	U	U	U	**Y**
Nelson et al. (2011)(42)	L	U	L	L	U	L	**N**
Norton et al. (2014)(43)	L	L	L	U	L	U	**N**
Percac-Lima et al. (2014)(44)	U	U	U	U	U	U	**Y**
Perron et al. (2013)(45)	L	U	U	L	U	U	**Y**

Note: Asterisk denotes that the study was included in primary and secondary meta-analyses
Key: L= low risk, H= high risk, U= Unclear risk.

Appendix 7: Funnel plots

[Figure 4 near here]

[Figure 5 near here]
